# Optimizing Few-Shot Learning Based on Variational Autoencoders

**DOI:** 10.3390/e23111390

**Published:** 2021-10-24

**Authors:** Ruoqi Wei, Ausif Mahmood

**Affiliations:** Department of Computer Science & Engineering, University of Bridgeport, Bridgeport, CT 06604, USA; ruoqiwei@my.bridgeport.edu

**Keywords:** deep learning, variational autoencoders, data representation learning, generative models, unsupervised learning, few shot learning, latent space, transfer learning

## Abstract

Despite the importance of few-shot learning, the lack of labeled training data in the real world makes it extremely challenging for existing machine learning methods because this limited dataset does not well represent the data variance. In this research, we suggest employing a generative approach using variational autoencoders (VAEs), which can be used specifically to optimize few-shot learning tasks by generating new samples with more intra-class variations on the Labeled Faces in the Wild (LFW) dataset. The purpose of our research is to increase the size of the training dataset using various methods to improve the accuracy and robustness of the few-shot face recognition. Specifically, we employ the VAE generator to increase the size of the training dataset, including the basic and the novel sets while utilizing transfer learning as the backend. Based on extensive experimental research, we analyze various data augmentation methods to observe how each method affects the accuracy of face recognition. The face generation method based on VAEs with perceptual loss can effectively improve the recognition accuracy rate to 96.47% using both the base and the novel sets.

## 1. Introduction

The explosion of big data has provided enough training samples in the real world to facilitate the development of deep learning performance [1,2,3]. Moreover, as a result of the development of high-performance computing devices such as graphics processing units (GPUs) and CPU clusters in recent years, the training of large-scale deep learning models has been greatly improved for big data feature learning. Compared to the early K80 (2014) GPU, which has 13 streaming microprocessors (SMs) with 2496 CUDA cores, NVIDIA’s latest flagship GPU A100 (2020) has 108 SMs with 6912 CUDA cores, resulting in an approximately 10-fold faster novel performance for deep learning [4,5]. At present, deep learning models are often successful with millions of model parameters and a large amount of labeled big data available for training. Deep learning has also fueled significant progress in a variety of computer vision problems, such as object detection [6,7,8,9], motion tracking [10,11,12,13], action recognition [14,15], human pose estimation [16,17,18,19], and semantic segmentation [20,21,22,23]. Great success has also been achieved in face recognition with convolutional neural networks (CNN) [24,25,26,27,28,29]. However, many applications of this kind of deep learning in face recognition can only be realized on the premise of having a large amount of labeled data. Moreover, in real life, due to restrictions such as data security management and labor costs, it is impractical to obtain such a large amount of labeled data.

Humans, after learning only a few images of a target, can recognize and, sometimes, can even perceptually recognize the same target without learning the target image. Inspired by the ability of humans to learn quickly from a small number of samples, the field of artificial intelligence (AI) is currently actively researching few-shot learning, to solve the problems caused by limited datasets, by imitating the process of rapid recognition of the human brain to bring AI applications closer to the actual real-world scene.

The purpose of few-shot learning (FSL) is to learn the classifier of new classes; each class provides only a few training examples [30,31,32]. For instance, regarding practical applications of face recognition, such as surveillance and security, the face recognition system should be able to recognize people who have only seen it a few times; that is, the machine has the ability to see and understand things in the same manner as humans. Among the few existing sample-learning methods, data augmentation is an important approach. It is a weakly supervised series of techniques aimed at expanding datasets with additional data points. However, it is very challenging for existing machine learning methods because this limited dataset does not represent the data variance which describes the degree of spread in the dataset. Unbalanced data distribution or lack of data will cause over-parameterization and over-fitting problems, resulting in a significant decrease in the effectiveness of deep learning results. In face recognition, in particular, the variance in facial attributes, such as wearing glasses, which may detract from the recognition of the eye region and the overall facial appearance. Another example is wearing a beard, which makes it difficult to capture the boundary of the face and other features around the jawline, and can cause significant intra-class differences between faces of the same person, thus seriously affecting face recognition performance. To solve these problems, the construction of deep generative models has been attempted to refine the data and to convert the original data into features, thereby increasing the intra-class variations of the dataset. The most recent deep generative networks are VAEs [33,34,35] and generative adversarial networks (GANs) [36]. VAEs do not suffer from the problems encountered in GANs, which mainly relate to nonconvergence causing mode collapse and difficulty of evaluation [36,37,38]. In addition, a key benefit of VAEs is the ability to control the distribution of the latent representation vector *z,* which can combine VAEs with representation learning to further improve the downstream tasks [35,39]. VAEs can learn the smooth latent representations of the input data [40] and can thus generate new meaningful samples in an unsupervised manner.

In this research, we attempted to apply the VAE to the few-shot learning problem due to the scarcity of labeled training data. We employed the architecture proposed by [41] to train a model with a base set based on transfer learning and then build a feature extractor. Then, we undertook fine-tuning to learn the actual label of the target using a novel image dataset from the data augmentation. A face dataset is divided into a base set and a one-shot set. The base set implies that each person has only one picture. The one-shot set also means each person has only one picture. It is important to note that there is no overlap between the base set and the one-shot set. Using transfer learning as the backend, we implemented various types of data generation to increase the intra-class variations of the base set, thereby achieving higher recognition accuracy. Our face data augmentation for few-shot learning based on VAEs is fundamentally important for improving the performance of neural networks in the following aspects: (1) It is inexpensive to generate a huge number of synthetic data points with annotations in comparison to collecting and labeling real data. (2) Synthetic data can be accurate, so it is consistent with the ground-truth by nature. (3) If controllable generation method is adopted, faces with specific features and attributes can be obtained. (4) Face data augmentation has some special advantages, such as generating faces without self-occlusion [42] and a balanced dataset with more intra-class variations [43].

The main contributions of our work include:

- We employ a generative approach using variational autoencoders (VAEs) to optimize few-shot learning tasks by generating new samples with more intra-class variations.
- We analyze various data augmentation methods to observe how each method affects the accuracy of face recognition.
- We can generate synthetic data which is accurate and consistent with the ground-truth by nature, balance the dataset, and manipulate specific face attributes of the generated faces.
- Our framework significantly improves the face recognition accuracy rate in few-shot learning scenarios (up to 96.47% for the LFW dataset).

The structure of our paper is as follows. Section 2 describes background studies to our research. Section 3 explains the research plan in detail: (1) proposed architecture overview, (2) deep convolutional networks, (3) generation networks, (4) verification networks, and (5) identification networks. Section 4 outlines the experiments with implementation details and results. The summary, conclusion, and future work are provided in Section 5.

## 2. Related Work

### 2.1. Few-Shot Learning

Few-shot learning was proposed to solve the problem of learning new classes in classifiers, where each class provides only a small number of training samples [30,31,32]. As a result of the development of deep learning techniques, the existing FSL methods can in the following: metric learning [44,45,46,47], which learns metrics/similarity of few-shot samples through deep networks; meta-learning [48,49,50], which learns a meta-model in multiple FSL tasks, and then the meta-model can be used to predict the weight of the model in a new FSL task; transfer learning [51,52], which uses pretrained weights as initialization; and data augmentation [53,54], which is a form of weak supervision [55] and aims to expand the few-shot sample dataset with additional data points. It should be noted that there is no absolute distinction between the four categories. In this paper, our idea utilizes triplet-loss-based metric learning for few-shot face verification, utilizes data augmentation to improve the few-shot face recognition, and builds upon the transfer learning backend.

### 2.2. Data Augmentation

Data augmentation [53,54,56] is a technique used to increase the amount of available training data. The simplest method in data augmentation is the expansion of the dataset through basic digital image processing [57]. Digital image processing [58] includes photometric transformations [59] and geometric transformations [60]. Image processing is a traditional but powerful image augmentation method. However, these methods generate only repeated versions of the original data, and the dataset lacks intra-class variations. Therefore, its application is primarily to uniformly transform the entire image, rather than transforming specific attributes of the face. 

Model-based face data augmentation is used to fit a face model to the input face, and then generate faces with different attributes by changing the parameters of the fixed model. Commonly used model-based face data augmentation techniques can be divided into 2D active appearance models (2D AAMs) [61] and 3D morphable models (3D MMs) [62]. Compared to image processing, the model-based method can be used to generate intra-class variations such as pose transformation and expressions. However, one of the biggest challenges is the difficulty in generating the teeth and mouth of the human face because these models can only generate the surface of the skin, and not the eyes, teeth, and mouth cavity [53]. Another shortcoming is that when the head posture changes, the lack of occlusion area causes artifacts [54]. Therefore, it is difficult to reconstruct a complete and accurate face model from a single 2D image through model-based face data augmentation. In addition, the computation cost of this method is also very high. In this research, our generative model-based transformation method does not only deal with pose transformation and expression transfer, but also with realistic facial attributes transformation at an affordable cost.

### 2.3. Generative Models

The principle of the deep generative model in the generation of new data is to use distribution estimation and sampling [38,63]. The traditional deep generative model is the Boltzmann series, that is, deep belief networks (DBNs) [64] and deep Boltzmann machines (DBMs) [65]. However, one of their main limitations is the high computational cost during the operation process [3]. The latest deep generative networks are VAEs [33,34,35] and generative adversarial networks (GANs) [36,66]. VAEs do not suffer problems encountered in GANs, which are mainly nonconvergence causing mode collapse and difficulty of evaluation [36,37,38]. In addition, a key benefit of VAEs is the ability to control the distribution of the latent representation vector *z,* which can combine VAEs with representation learning to further improve the downstream tasks [35,39]. VAEs can learn the smooth latent representations of the input data [40] and can thus generate new meaningful samples in an unsupervised manner. These properties have allowed VAEs to enjoy success, especially in computer vision; for example, static image generation [67,68], zero-shot learning [69,70,71], image super-resolution [72,73], network intrusion detection [74,75,76], and semantic image inpainting [77,78]. Table 1 lists the advantages and disadvantages of the proposed VAE data augmentation method along with those of other methods. However, compared with GANs, the samples generated by VAEs tend to be blurred and of lower quality. Recently, a breakthrough was made in VAEs by employing a perceptual loss function instead of reconstruction loss. The perceptual loss function is based on the high-level features extracted from pretrained deep CNNs to train feed-forward networks. It captures perceptual differences and spatial correlations between output and ground-truth images and solves the problem of blurry figures, thus resulting in high image quality [79,80]. Therefore, in our research, we employ VAEs using perceptual loss to generate networks.

### 2.4. Transfer Learning

The methods of transfer learning [81] can be divided into inductive transfer learning, transductive transfer learning, and unsupervised transfer learning. Furthermore, the inductive transfer learning method can be summarized into four situations based on the “what to transfer”. Among these, instance-based transfer learning refers to reweighting samples in the source domain and correcting the marginal distribution difference between it and the target domain. This method works best when the conditional distributions in the two domains are the same. Feature-representation transfer is suitable for homogeneous and heterogeneous problems. This method works best when the source and the target domains have the same label space. Parameter-transfer approach transfers knowledge through the shared parameters of the source and the target domain learner models. As the pretrained model on the source domain has learned a well-defined structure, the pretrained model can be transferred to the target model if the two tasks are related. Because fine-tuning requires much less labeled data, this approach can potentially save time, reduce costs, and help improve robustness. The basic assumption of the relational knowledge-transfer problem [50] is that there are some common relations between the data in the source and the target domains. Therefore, the knowledge to be transferred is the common relationship between the source and the target domains. In this research, we focus on parameter-transfer approach-based transfer learning. This means that fine-tuning the CNN parameters from a pretrained model using a target training dataset is a particular form of transfer learning. 

### 2.5. Facial Attribute Manipulation

Facial attribute analysis [82,83] includes facial attribute estimation (FAE), which is used to identify whether there are facial attributes in a given image, and facial attribute manipulation (FAM), which is used to synthesize or remove specific facial attributes [84]. In this research, we focus on FAM. There are two main methods to undertake FAM using generative models: model-based methods and extra-condition-based methods [84]. Model-based methods can only edit an attribute during a training process, but the disadvantage is its significant computation costs. Furthermore, there are two kinds of extra-condition-based methods: (1) attribute vectors as extra conditions, which, with an extra input vector, rely on simple linear interpolation; and (2) reference exemplars as extra conditions, which directly learn the image-to-image translation along with attributes, and is a popular approach for unsupervised disentanglement learning. However, disentangling is not easy to achieve, and to obtain better disentangling, the quality of reconstruction must be sacrificed [85]. Therefore, in this research, we focus on the first method that takes an attribute vector as the guidance to manipulate the desired attribute. Specifically, by changing a specific face attribute vector, the attributes of the face can be updated accordingly, referred to as deep feature interpolation (DFI) [86].

## 3. Research Plan

### 3.1. Proposed Architecture Overview

In this study, we analyzed the data augmentation method based on a variational autoencoder (VAE) in order to improve the accuracy and robustness of few-shot face recognition. We also increased the size of the training dataset in various ways to observe how data augmentation affects identification accuracy.

The complete deep face recognition system can be divided into three modules [87]: (1) a face detector to locate faces in images, (2) a facial landmark detector that can align faces with normalized coordinates, and (3) the face recognition module. We only focus on the face recognition module throughout the remainder of this paper.

Furthermore, face recognition can be divided into face verification (which answers the question, is this the same person?) and face identification (which addresses the question, who is this person?) [41]. Face verification calculates one-to-one similarity to determine whether two images belong to the same face, whereas face recognition calculates one-to-many similarities to determine the specific identity of the face. The face recognition module includes the following processes: (1) face processing, (2) deep feature extraction, and (3) face matching or face identification. In this research, we present the data augmentation method, which is facial attribute manipulation (FAM) using VAEs. Second, we adopt a pretrained architecture of Inception ResNet v1 [88] as a CNN for deep feature extraction for face data augmentation, face verification, and face identification tasks.

**Figure 1:**
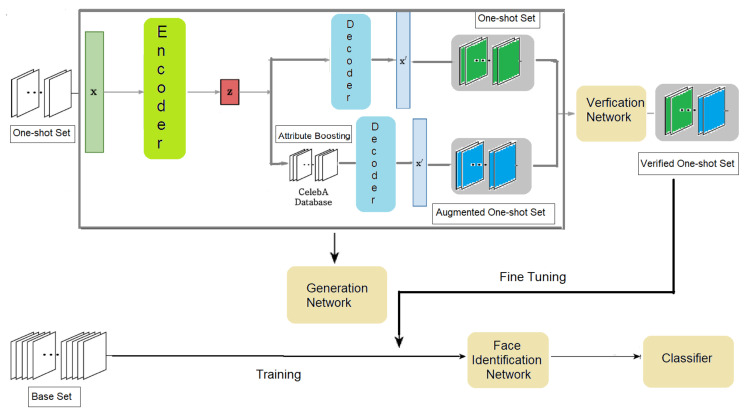
Proposed architecture overview.

The proposed idea is as follows (as shown in Figure 1): a face dataset is divided into a base set and one-shot set. The base set means that each person has just one picture. The one-shot set also means that each person has only one picture. It should be noted that there is no overlap between the basic set and the one-shot set. Although the pictures in the base set and the one-shot set belong to the same person, the difference is that there may be changes in expression or posture between them, so they may not be correctly recognized. The face identification network is first pre-trained on the base set, and then fine-tuned through the augmented one-shot set. However, the face identification accuracy may not be good enough. Because the accuracy of individual recognition is proportional to the number of training images for each person, we can increase the accuracy of the one-shot set by adding augmented data. Thus, we used the proposed data augmentation method to increase the one-shot set and studied the change in the identification accuracy by this method. However, the problem with this method is that the performance of face identification may decrease with several augmented data points because the identity information is not sufficient. This is particularly the case for those images generated by VAEs. In order to solve this problem, we use a verification network to filter images that cannot be recognized; that is, if the augmented data can successfully pass the verification network, we pick out a subset from it. Finally, we can use these qualified augmented one-shot sets to fine-tune the face identification network with the original base set and the one-shot set. We hope to use this method and transfer learning as the backend to achieve higher accuracy of few-shot face recognition.

### 3.2. Deep Convolutional Networks

**Figure 2:**
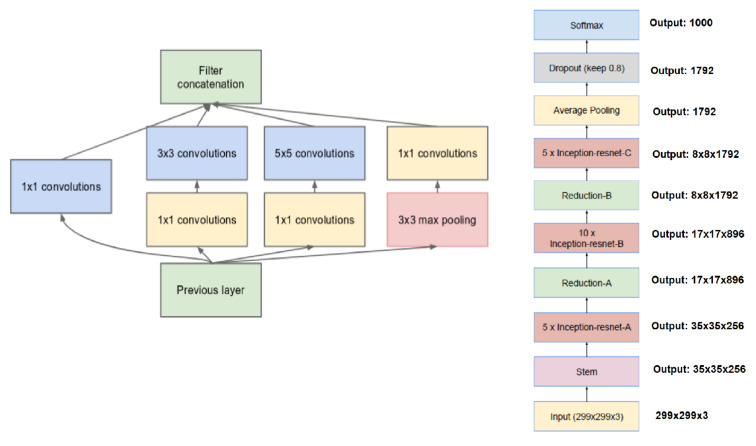
Inception module with dimension reductions (left) and schema for Inception-ResNet-v1 (right).

We chose the best architecture for the results of the Computation Accuracy Tradeoff [41]—Inception Resnet V1—as the Deep Convolutional Network for the FaceNet system (as shown in Figure 2).

This deep neural network is almost the same as that described in [89]. The main difference between them is that the *L_2_* pooling is used in a local specific area instead of the maximum pooling (m). The pooling layer can reduce the number of parameters in the subsequent operation. The idea of *L_2_* pooling is to use *L_2_* regularized for pixel values in a local specific area, i.e., except for the final average pooling, the pooling is always 3 × 3 and is parallel to the convolution modules in each Inception module. If the dimensionality is reduced after pooling, it is represented by p. We then utilize 1 × 1, 3 × 3, and 5 × 5 pooling to concatenate and obtain the final output. Figure 2 describes the CNN network in detail. Note that all of our specific networks described in the next sections are based on this CNN framework.

### 3.3. Generation Network

In our research, we employ VAE using FaceNet-based [41] perceptual loss similar to that in the paper [90] for face image generation with boosting attributes. Specifically, the pixel-by-pixel reconstruction loss of the deep convolutional VAE is replaced by a feature perceptual loss based on a pre-trained deep CNN. The feature perceptual loss is used to calculate the difference between the hidden representations of two images extracted from a pretrained deep CNN such as AlexNet [91] and VGGNet [92] trained on ImageNet [27]. This method attempts to improve the quality of the image generated by the VAE by ensuring the consistency of the hidden representation of the input image and the output image. It also imposes the spatial correlation consistency of the two images. The generative model consists of two parts—one is the autoencoder network, which includes an encoder network *E(x)* and a decoder network *D(z),* and the other is a pre-trained deep CNN, which is used to calculate the feature perceptual loss network *ϕ*. The encoder maps an input image *x* to a latent vector *z* = *E(x)*; then, the decoder maps the latent vector *z* back to image or data space *x’ = D(z*). After the VAE is trained, the decoder network can use the given vector *z* to generate a new image. We need two loss functions to train the VAE. First, after encoding an image *x* to a latent vector $z=Encoder\left(x\right)\;\sim \;q(z|x)\;,$ the difference between the distribution of  q(z|x) and the distribution of a Gaussian distribution (called KL Divergence) can be minimized by the gradient descent algorithm: the first is KL divergence loss $L_{KL}=D_{kl}[q(z|x)||p\left(z\right)]$ [33] which is used to ensure that the latent vector *z* is a Gaussian random variable. The other is feature perceptual loss, which computes the difference between hidden layer representations, i.e., $L_{Rec}=L_{1}+L_{2}+\ldots +L_{n}$, where $L_{n}$ is the feature loss at the *n*th hidden layer. During the training process, the pre-trained CNN network is fixed and is only used for advanced feature extraction. KL divergence loss $L_{kl}$ is only used to update the encoder network, and feature perception loss $L_{Rec}$ is used to update the encoder and decoder parameters.

#### 3.3.1. Variational Autoencoder Network Architecture

**Figure 3:**
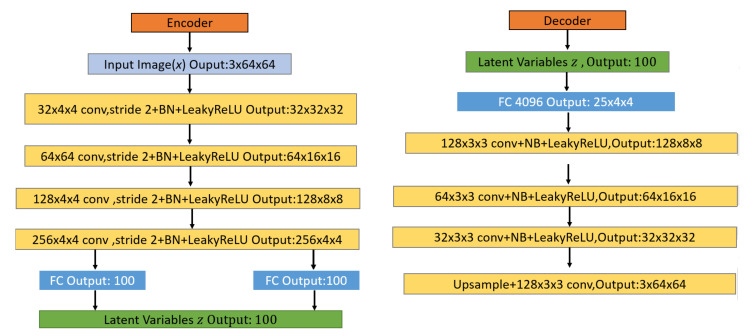
VAE architecture for the encoder network (left) and the decoder network (right).

The neural networks of the encoder and decoder are both constructed from deep CNN models such as AlexNet [91] and VGGNet [92]. As shown in Figure 3, this structure includes fully connected (FC) layers and convolutional (Conv) layers. The input image passes through four Conv layers and the last FC layer until the latent variable space is reached. The two convolutional layers in the encoder network achieve feature maps’ dimensionality reduction using a stride of 2 and a 4 × 4 kernel size, which refers to the size of the filter that encodes a specific feature. After each Conv layer, there is a batch normalization layer and a LeakyReLU activation layer. Finally, two fully connected output layers (for *μ* and *σ^2^*) are used to calculate KL divergence loss and sample latent variable *z*. The generative model$\;p\left(x|z\right)$ takes the sampled latent variables z received by μ and *σ^2^* and, using the reparameterization trick, feeds it through one FC layers and four Conv layers until a reconstructed output is obtained. Finally, it uses a stride of 1 and a kernel size of 3 × 3 in the deconvolution to obtain the reconstruction image. For upsampling, compared to the fractional-strided convolutions used in other works [23,63], we use the nearest neighbor method at a scale of 2. After each Conv layer, there is also a batch normalization layer and a LeakyReLU activation layer to help stabilize training.

#### 3.3.2. Feature Perceptual Loss

**Figure 4:**
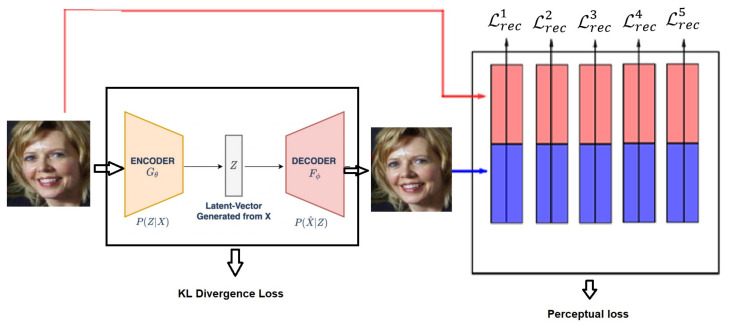
VAE with perceptual loss architecture overview.

The feature perception loss of two images is defined as the difference between hidden representations in the pre-trained deep CNN *ϕ.* We use Inception ResNet V1 as the Deep Convolutional Network for the FaceNet system in our experiment (as shown in Figure 4).

(1) $$L_{rec}^{n}=\frac{1}{2C^{n}\;\times W^{n}\times H^{n}}\overset{C^{n}}{\underset{c=1}{\sum }}\overset{W^{n}}{\underset{w=1}{\sum }}\overset{H^{n}}{\underset{h=1}{\sum }}{\left(\phi {\left(x\right)}_{c,w,h}^{n}-\phi {\left(x^{\prime }\right)}_{c,w,h}^{n}\right)}^{2}$$

The final reconstruction loss is the total loss obtained by adding the losses of the different layers of the deep CNN network, that is $L_{rec}$ = $\sum_{n}L_{rec}^{n}.$ In addition, we must add KL divergence loss ${}_{kl}$ to ensure that the latent vector z is a Gaussian random variable. Therefore, the training mode of this VAE model is to jointly minimize. 

The KL divergence loss $L_{kl}$ and the total feature perceptual loss $L_{rec}^{n}$ of the deep CNN network; that is,

(2) $$L_{total}=\alpha_{kl}\;+\beta \overset{n}{\underset{i}{\sum }}L_{rec}^{n}$$

where α and β are weighting parameters for KL divergence and feature perceptual loss [93].

#### 3.3.3. Attribute Boosting using VAEs

In order to be able to manipulate attributes such as face attributes and gender, the attribute vector needs to be calculated first, which can be achieved through simple vector arithmetic operations [94], thereby showing a rich linear structure in the representation space. A well-known example of vector arithmetic operations is vector(‘King’)-vector(‘Man’) + vector(‘Woman’) resulting in a Queen’s vector. In this study, we performed a similar arithmetic on the *Z* representation of our generators for visual concepts. In this paper, we investigate facial attributes of smiling. This is done by finding all images where the attribute ’Smiling’ is not present and where the same attribute is present. The attribute vector is then calculated as the difference between the two average latent variables. Specifically, the images of two different attributes are sent to the encoder network to calculate the latent vectors, and the average latent vectors are calculated for each attribute, and are represented as *z_pos_smiling_* and *z_neg_smiling_*. We can then use the difference *z_pos_smiling_ − z_neg_smiling_* as the latent vector *z_smiling_* of the smiling attribute. Finally, applying this smiling attribute latent vector to different latent vectors *z* to calculate a new latent vector *z*, such as *z* + *z_smiling_*, where = 0, 0.1 … 1, and then feeding the new latent vector *z* to the decoder network generates new face images.

### 3.4. Verification Network

**Figure 5:**
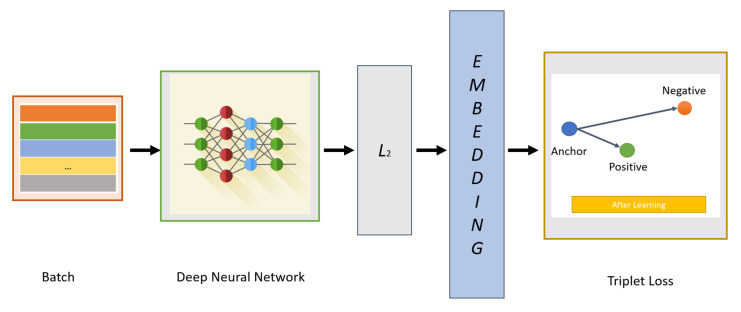
Face verification network architecture.

After generating new faces with specific attributes, we can select the new faces if they can successfully pass the verification network. The verification network also uses the CNN architecture of FaceNet [41] (as shown in Figure 5). The loss function we use in the face verification model is the triplet loss; that is, we embed *f(x)* from the image *x* into the feature space R^d^, and then minimize the squared distance between faces from the same identity and maximize the squared distance between faces from different identities [95].

The following sections describe: (1) learning face embedding with triplet loss; (2) triplet selection; and (3) the face verification task.

#### 3.4.1. Learning Face Embedding with Triplet Loss

**Figure 6:**
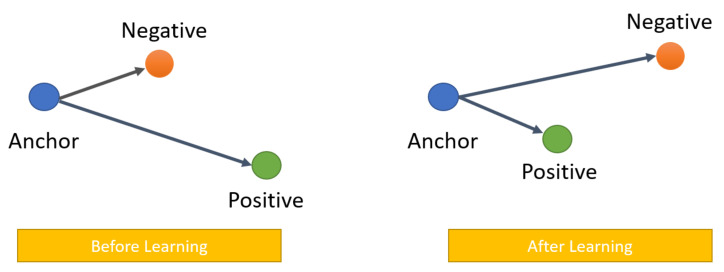
Triplet loss overview.

The idea of face embedding in this section is to embed the image *x* into a d-dimensional Euclidean space as *f(x)* ∈ R^d^. It should be noted that this embedding is limited to the d-dimensional hypersphere, that is, the *L*_2_ norm||*f(x_)_*||_2_ = 1. This loss is caused by nearest neighbor classification [96]. Here, the network is trained to ensure that the output distance between an image $x_{i}^{a}$ (anchor) and all other images $x_{i}^{p}$ (positive) that belong to a known person is as close as possible, and that the distance between an image $x_{i}^{a}$ (anchor) and any image$\;x_{i}^{n}$ (negative) that belongs to an unknown person is as distant as possible (as shown in Figure 6). Thus,

(3) $${\left|\left|f\left(x_{i}^{a}\;\right)-f\left(x_{i}^{p}\right)\right|\right|}_{2}^{2}+\alpha <{\left|\left|f\left(x_{i}^{a}\;\right)-f\left(x_{i}^{n}\right)\right|\right|}_{2}^{2},\;\forall ((f\left(x_{i}^{a}\;\right),f\left(x_{i}^{p}\right),f\left(x_{i}^{n}\right)\in T$$

where the threshold α is a margin that is enforced between positive and negative pairs. *T* is the set of all possible triplets. Then, the triplet loss is as follows:

(4) $$L=\overset{N}{\underset{i}{\sum }}[{\left|\left|f\left(x_{i}^{a}\;\right)-f\left(x_{i}^{p}\right)\right|\right|}_{2}^{2}-{\left|\left|f\left(x_{i}^{a}\;\right)-f\left(x_{i}^{n}\right)\right|\right|}_{2}^{2}+\alpha ]$$

#### 3.4.2. Triplet Selection

There are two ways to achieve the selection of triplets, as follows:

1. Offline triplet mining: For example, at the beginning of each epoch, we calculate all embeddings on the training set, and then select only hard or semi-hard triples. Then, we can train the epoch with these triplets. However, this technique is not very effective because we need a complete pass of the training set to generate triplets. It also requires regular updates of triplets offline.
2. Online triplet mining: The idea here is to dynamically calculate useful triples for each batch of input. This technique allows you to provide more triples for a single batch of input without any offline mining and is more efficient. Therefore, we focus on online triplet mining. For example, if we want to generate triplets from these *B* embeddings, whenever we have three indexes *i, j, k* ∈ [1, *B*], if examples *i* and *j* have the same label but different images, and example k has different labels, then we say (*i, j, k*) is a valid triple.

#### 3.4.3. Face Verification Task

After learning the embedding using triplet loss with FaceNet’s CNN, we can utilize this embedding for the FaceNet verification tasks. For example, the input is the paired faces with smiling and unsmiling faces, and the output is the *L*_2_ distance through the verification network between the paired faces. If their *L*_2_ distance is less than 1.1 [41], the pair of images are from the same person, otherwise they are not.

### 3.5. Identification Network

Finally, we can use these qualified augmented one-shot sets to fine-tune the face identification network with the original base set and the one-shot set. Similar to the verification network, our face identification network also combines the architecture and training strategy of the FaceNet with the deep convolutional networks and Softmax classifier.

The Softmax function takes a vector *z* of K real numbers as input and normalizes it to a probability distribution consisting of K probabilities that are proportional to the exponent of the input number. Before the *z* vector component is input to Softmax, the input *z* will not be in the interval (0,1). After Softmax is applied, however, each component will be in the interval (0,1), and the sum of the components is 1, so Softmax can convert the input *z* into a probability.

The Softmax function is defined by the formula:

(5) $$\sigma {\left(z\right)}_{i}=\frac{e^{z_{i}}}{\sum_{j=1}^{K}e^{z_{i}}}$$

for *i* = 1,…, *K* and z = (*z_1_,…, z_K_*)∈R*^K^*.

## 4. Experiments and Results

### 4.1. Datasets

We used the dataset of Labeled Faces in the Wild (LFW) [97] for training face identification network. The LFW dataset contains 13,233 face images with a total of 5749 identity labelled faces [98]. Among these, 1680 people have two or more images. The generative model VAE is trained by the CASIA-WebFace dataset [99], which has a total of 10,575 people and a total of 494,414 images. In the attribute boosting process, the CelebFaces Attributes Dataset (CelebA) [100] is utilized to extract attribute variables vectors. There are about 10k people in total, including 202,599 face images, and each image has 40 binary attribute annotations. Animals’ faces (for example, dogs) can also be processed by the Deep Neural Networks. However, the size of the animals’ face database will be enhanced and different conditions may be considered during the acquisition of an animal image for each subject: pose variation, distance variation, illumination variation, and occlusion (covering, non-covering) variation. Moreover, the currently available popular large-scale face recognition databases are all based on adults and there is no version for children; hence, our experiment is based on adults.

### 4.2. Implementation Details

#### 4.2.1. Data Preprocessing and Face Alignment

Before the start of each training phase, each image data must be pre-processed to adapt to the face alignment network. We crop the rectangular image into a square with the side length of the short side of the original image. Then, we roughly align the image according to the eye position and adjust the image to 224 × 224-pixel RGB images.

The Multi-task Cascaded Convolutional Network (MTCNN) is a face detection and alignment method based on deep convolutional neural networks. This method can be used to complete face detection and alignment tasks at the same time. Pre-processing the images into 224 × 224-pixel RGB images is required before using the MTCNN. All face images are then detected, five key points are utilized to align the face images, and finally, all images are cropped and uniformly scaled to 160 × 160-RGB pixels.

#### 4.2.2. Face Generation Using VAE

This section describes how to reconstruct face images with boosting attributes by the VAE using FaceNet-based perceptual loss.

(a) Train a VAE: This section describes how to train a VAE using perceptual loss. The VAE generation model is trained on the CASIA-WebFace Dataset. We use a batch size of 128 and 50,000 epochs to train the VAE model, and the NAdam method used to optimize [101] the initial learning rate is 0.01. The Inception ResNet CNN is used as the loss network *ϕ* to calculate the feature perception loss for image reconstruction. The size of the generated images is decided by the VAE implementation that generates 64 × 64-pixel images. The hardware specifications for executing implementations are a Tesla P100 GPU with 25 GB RAM. Table 2 shows some values of hyper-parameters used in this experiment.
(b) Calculate Attribute Vectors: In this step, the CelebA dataset is used to calculate vectors in latent variable space for several attributes. The CelebA dataset contains ~200 k images annotated with 40 different attributes such as Blond Hair and Mustache. This is done by finding all images where the attribute is not present and where the same attribute is present. The attribute vector is then calculated as the difference between the two average latent variables. The VAE model checkpoint should point to the checkpoint trained in Step 1. Before running this, the CelebA dataset should also be aligned. The list_attr_celeba.txt file of the CelebA dataset contains the 40 attributes for each image and is available for download together with the dataset itself.
(c) Attribute Boosting Using VAEs: To demonstrate the usage of the VAE, after which we can modify attributes of an image, we apply the attribute-specific vector (calculated in step b) to different latent vectors *z* to calculate a new latent vector *z*, such as *z* + *z_attribute_*, where α = 0, 0.1 … 1, and then feed the new latent vector *z* to the decoder network to generate new face images. Specifically, we select a few faces in the one-shot set where the specific attribute is not present. We then feed these images to the encoder of VAE, and then calculate the latent variables. We then add different amounts of the specific attribute vector (calculated in Step b) to different latent variables *z* and generate new faces with specific attribute images.

#### 4.2.3. Face Verification and Face Identification Experiments

The face verification function under the deepface interface offers to verify face pairs as the same person or different persons. This is a pre-trained network so there is no need to retrain. Table 2 shows some values of hyper-parameters to train the Softmax classifier for the Face Identification Network, which were used in all experiments. For comparison purposes, some parameters for all experiments were set to the same values to perform a fair comparison.

### 4.3. Results

#### 4.3.1. Quality of The Reconstruction

**Figure 7:**
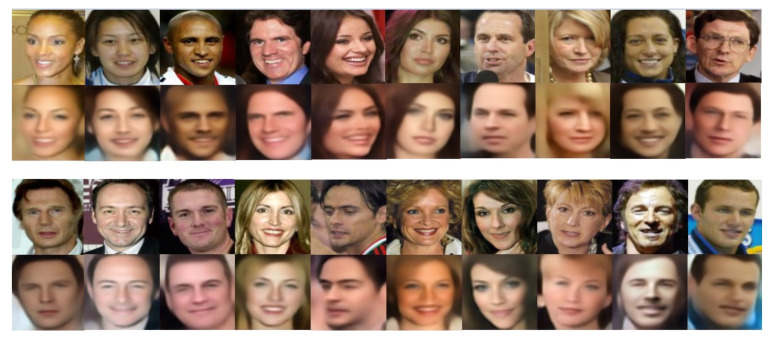
Face reconstruction by VAE with feature perception loss. Top row: Input images. Bottom row: Generated images from VAE with feature perceptual loss.

In this research, we first used VAE based on feature perception loss to reconstruct face images. Figure 7 shows the result of the reconstruction. Top row: Input images. Bottom row: Generated images from VAE with feature perceptual loss. As shown in Figure 7, we can observe the difference between the face reconstructed by the VAE based on the feature perception loss and the original input face: the VAE based on the feature perception loss can not only generate human-like faces and the reconstructed face is similar to the original input face, but it can preserve the overall spatial face structure. We know that it is difficult for ordinary VAE to generate clear facial parts, such as eyes, nose, and mouth. This is because ordinary VAE tries to minimize the pixel-by-pixel loss between two images, whereas pixel-based loss does not contain perceptual and spatially related information. However, VAE based on the loss of feature perception can generate clear facial parts, such as eyes, nose, and mouth. This point of view is also confirmed in our experiments. Note that a too bright background will reduce the quality of the dark field on a face, thereby decreasing the quality of the reconstruction. When one captures images in low-light conditions, the images often suffer from low visibility. Moreover, images captured in low-light conditions usually suffer from very low contrast, which increases the difficulty of subsequent computer vision tasks to a great extent.

#### 4.3.2. Pose Transition

**Figure 8:**
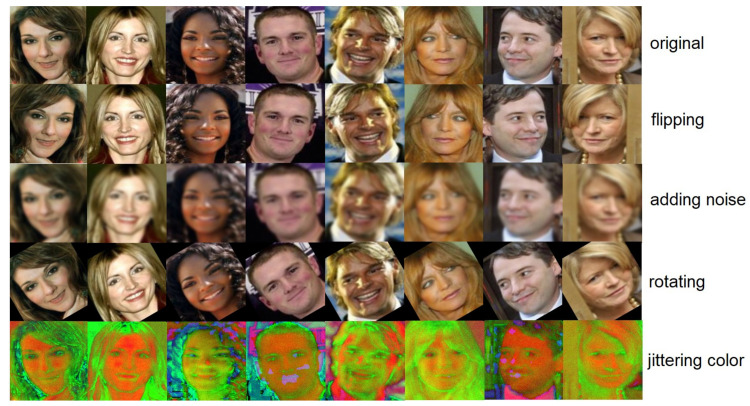
Face augmented data by modifying images via rotating, flipping, adding noise, and jittering color.

In this experiment, we also tried to augment data by modifying images via rotating, flipping, adding noise, and jittering color. Our model includes these conventional schemes to provide prevention of overfitting that can occur within an inter-class, increase robustness, and achieve higher scores. The effectiveness of classical data augmentation is empirically shown in several previous studies. As shown in Figure 8, the first row is the original face, the second row is the augmented data by flipping, the third and the fourth rows are the results of adding noise and rotating, respectively, and the last row shows the augmented data by jittering color. In this way, each person has four different pose transitions; thus, in this step of the experiment, each person generates a total of four new pictures by basic image processing.

#### 4.3.3. Attribute Manipulation

**Figure 9:**
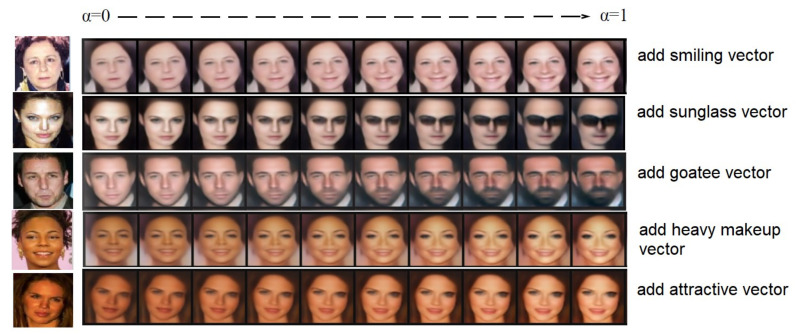
The vector arithmetic for visual attributes.

In this experiment, we did not seek to manipulate the overall face image, but aimed to control the specific attributes of the face image and generate a face image with specific attributes. By adding the vector of attributes to the face in the latent variable space, we can obtain the smooth transition process of adding this attribute to the face. As shown in Figure 9 (first row), by adding a smiling vector to the latent vector of non-smiling women, we can obtain a smooth transition from a non-smiling face to a smiling face. When the factor α increases, the appearance of the smile becomes more obvious, while other facial attributes can remain unchanged. Similarly, the second row is the transition process of adding a sunglasses vector from left to right, the third line and the fourth line are the results of adding a goatee vector and a heavy makeup vector, respectively, and the last line shows adding an attractive vector. In this manner, each person has 40 different attributes, and each attribute will generate 10 pictures. Thus, in this step of the experiment, each person will generate a total of 400 new pictures by VAE.

#### 4.3.4. Correlation between Attribute-Specific Vectors

**Figure 10:**
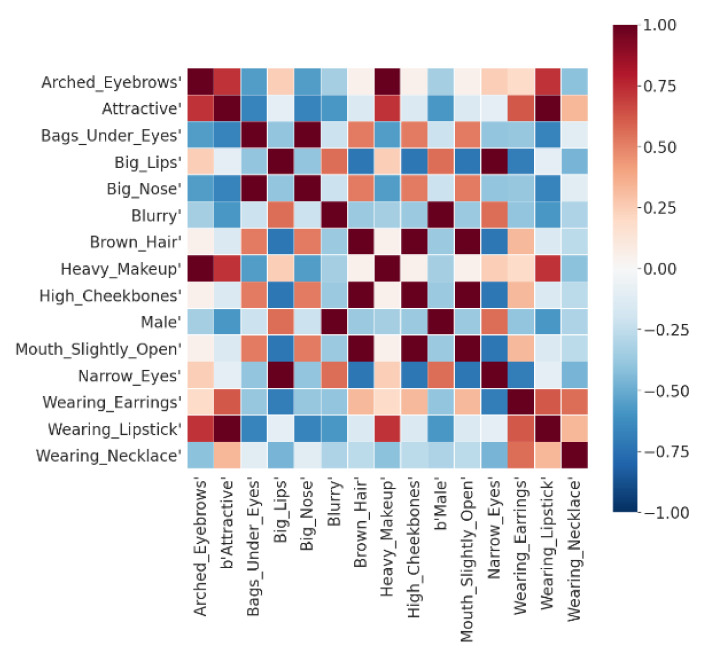
Pearson correlations between specific facial attributes.

There are often correlations between different facial attributes. For example, heavy makeup and lipstick are often related to women. In order to study the correlation between different facial attributes in the CelebA dataset, we selected 15 facial attributes and calculated their attribute-specific latent vectors. Then, we used Pearson correlation to calculate the correlation matrix of these 15 attribute-specific vectors. Figure 10 shows the weight visualization of the learned correlation matrix. Red indicates a positive correlation, blue indicates a negative correlation, and the intensity of the color indicates the strength of the correlation. From the visualization results, we can find many related attribute pairs and many mutually exclusive attribute pairs. For example, the attributes of arched eyebrows and heavy makeup are given relatively high weights, indicating a positive correlation between these two attributes. The attributes of arched eyebrows and male are given relatively low weights, indicating a negative correlation between these two attributes. It is expected that women are generally considered to use more cosmetics than men. In addition, wearing a necklace seems to have no correlation with most other attributes, and there is only a weak positive correlation between wearing necklace, wearing lipstick, and wearing earring. This can also be explained because wearing a necklace, lipstick, and earrings are all facial decorations that often appear together.

#### 4.3.5. Data Verification

Faces generated by deep generative models may occasionally fail to be recognized. For example, certain attributes (such as blond hair, young) added to the faces in the LFW dataset will result in a significant decrease in the face recognition rate. However, some attributes (such as pale skin, smiling) added to the faces in the LFW dataset can increase the face recognition rate. Therefore, to filter out unqualified augmented images and increase the face recognition rate, we used the verification network to verify the augmented data from the generation network, instead of manually selecting the appropriate attributes for the generation results. Table 3 shows the verification success rate of the generated images in the 1-shot experiment. At the same time, we also determined the verification success rate of the basic image processing data. It can be seen from the results that the verification rate of the basic image processing data is very high, further confirming that the use of basic image processing augmented data can enhance the robustness of our model and achieve higher scores. After this step, we can construct the final combined few-shot dataset: the novel image processing augmented dataset, the novel verified attribute augmented dataset, and the original basic dataset.

#### 4.3.6. Data Identification

Our final face identification network was pre-trained by using the basic set, and then fine-tuned using the novel image processing augmented dataset and the novel verified attribute augmented dataset, so that individuals in the no overlap one-shot test set can be recognized. For evaluation, we measured identification accuracy. That is, suppose there are N images available in the test set, and C images are correctly recognized. Then, the accuracy is defined as C/N. We propose two methods of data augmentation and a combination of these two methods: (1) basic image processing; (2) attribute boosting using VAE; and (3) the combination of the above two methods. In these three methods, we gradually add new data to study the changes in identification accuracy.

The results of face identification are shown in Table 4. From these experiments, we observe that before data augmentation, the basic one-shot set was used to train the face identification network and the no overlap one-shot test set was used for testing. The final face identification accuracy rate is 88.52%. This shows that there is still significant scope for improvement in the accuracy of one-shot face identification. The best accuracy of one-shot face identification using VAE attribute boosting augmentation is 92.99%, indicating that although using VAE attribute boosting augmentation can improve the performance of one-shot face identification, it still lacks robustness and causes overfitting that can occur within an interclass. By comparison, the best accuracy of one-shot face identification using basic image processing is 93.67%. This proves that basic image processing methods can prevent possible over-fitting between classes. In many applications, they can be combined to improve the performance of the model [91,102]. Therefore, our model can utilize these basic image.

Processing methods to prevent overfitting that can occur within an interclass, increase robustness, and achieve higher scores. We observe that the combination of basic image processing and attribute boosting using VAE is more effective in improving performance than using classic basic image processing and VAE attribute boosting. It should be noted that the identification accuracy increased from 92.99% and 93.67% to 96.47%, respectively. This result shows that the use of VAE to produce more diverse training faces makes up for the insufficiency of the intra-class variation. Table 5 shows that when the *L*_2_ distance of the verification network is set to less than 1.2 and 1.1 respectively, the results are sorted by *L*_2_ distance from small to large. Then, we pick top1 to top5 faces and add them to the training set, and observe the results of the effect of the added faces on the identification accuracy of the no overlap one-shot set. From the results, we can observe that no matter whether the distance of L2 is set to less than 1.2 or 1.1, the identification accuracy is always higher than the result before the data augmentation, which is 88.52%. This proves that our verified VAE attribute augmented set improved the accuracy of identifying the one-shot set. In addition, although the identification accuracy is higher when the L2 distance is set to 1.2 compared rather than 1.1, after the image processing augmented set is added, the accuracy of the L2 distance set to 1.1 is higher than that set to 1.2. This shows that setting the L2 distance to 1.1 makes the combination of the verified VAE attribute augmented set and image processing augmented set more effective, thereby improving the robustness and generalization ability of the face identification model.

## 5. Conclusions

At present, few-shot learning technology based on image generation is highly attractive in various computer vision applications, particularly because of the lack of labeled data during training. In this research, we attempted to use VAE with feature perception loss to generate better visual quality face images with boosting attributes, and expand the training set to improve the performance of the few-shot face identification task. As the size of the dataset increased, we verified the change in the performance associated with the increased use of Inception Resnet V1 for few-shot face recognition. With these results, we can generate accurate synthetic data that is consistent with the ground-truth, balance the dataset with more intra-class variations, and manipulate specific face attributes of the generated faces. Moreover, it is inexpensive to generate such synthetic data with annotations in comparison to collecting and labeling real data. Although our research was rigorously executed and verified to achieve sufficient results, there are some limitations to synthetic data generation, as described below.

Firstly, although our proposed method improved the identification accuracy of few-shot learning from 88.56% to 96.47%, which is a significant change, there is still room for improvement. One method to decipher the behavior of deep neural network classifiers is by investigating their decision boundaries and their geometrical properties. Deep neural network classifiers are vulnerable to erroneous instances near their decision boundaries [103]. Therefore, in the future, we plan to determine decision boundaries using adversarial examples between the different classes and then generate instances near the decision boundary to further improve the few-shot face identification accuracy. In addition, although the VAE with feature perception loss contains perceptual and spatially related information and can generate clear facial parts, the generated samples tend to be of lower quality compared to those of GANs. The VAE-GAN [104] is a strategy built on the VAE structure with a GAN discriminator added after the decoder, which ensures that the samples generated by the VAE have high quality. Therefore, in the future, we plan to utilize the VAE-GAN with feature perception loss to further improve the quality of the reconstruction. Furthermore, in this research, we focused on the method that takes an attribute vector as the guidance for manipulating the desired attribute. The advantage of this method lies in the manipulation of multiple attributes by changing multiple corresponding condition values. However, this method cannot continuously change a certain attribute because the value of the attribute vectors is discrete. We believe that, in the future, this limitation can be resolved through interpolation schemes [105] or semantic component decomposition [106].

Finally, reference exemplar-based algorithms based on unsupervised disentanglement learning [107,108,109] are becoming a promising research direction. Compared with only manually changing the attribute vector, this method directly learns the image-to-image translation along with the attributes, and then manipulates these attributes using a simple traversal across regularization dimensions, so that images with more realistic details can be generated. For our future research, we plan to incorporate unsupervised disentanglement learning in our framework.

## Figures and Tables

**Table 1:** Pros and cons of different data augmentation methods.

Methods	Pros	Cons
Basic Image Processing	Capable of Geometric Transformation such as rotation and flipping	Generate duplicated version of the original data
	Capable of Photometric Transformation such as color jittering and contrast adjustment	Lacks intra-class variations
Model-Based Transformation	Capable of generating 2D and 3D pose transformation	Only represents the skin surface and does not include eyes, teeth, and mouth cavity
	Synthesizes faces with different expressions	The artifacts caused by the missing of occluded regions when the head pose is changed
		Computationally expensive
Generative Adversarial Networks (GANs)	Capable of generating intra-class variations	Hard to evaluate
	Capable of generating high-quality image	Non-convergence causing mode collapse
Variational Autoencoders (VAEs)	Capable of learning smooth latent representations of the input data	The generated samples tend to be blurry compared to GANs.
	Decent theoretical guarantee	Posterior collapse

**Table 2:** Hyper-parameters used in all experiments.

HYPER-PARAMETERS	VALUES
**EPOCHS**	5000
**BATCH-SIZE**	128
**LEARNING RATE**	0.01
**OPTIMIZER**	Nadam
**DATASET**	LFW

**Table 3:** Data verification results.

	Verification Rate (%)
**Attribute boosting**	10
**Basic image processing**	98.3

**Table 4:** Face identification results.

Training Method	Test Set	Identification Accuracy (%)
**basic one-shot set**	no overlap one-shot set	88.52
**basic one-shot set + proposed verified VAE attribute augmented set**	no overlap one-shot set	92.99
**basic one-shot set + image processing augmented set**	no overlap one-shot set	93.67
**basic one-shot set + image processing augmented set + proposed verified VAE attribute augmented set**	no overlap one-shot set	96.47

**Table 5:** Face identification results with different *L*_2_ distances.

Training Method	Test Set	Identification Accuracy (%)				
		Top1-Shot	Top2-Shot	Top3-Shot	Top4-Shot	Top5-Shot
**basic one-shot set + proposed verified VAE attribute augmented set with 1.1 *L*_2_ distance**	no overlap one-shot set	89.60	90.36	**90.69**	90.36	90.27
**basic one-shot set + image processing augmented set+ proposed verified VAE attribute augmented set with 1.1 *L*_2_ distance**	no overlap one-shot set	95.70	95.52	95.67	**96.30**	95.36
**basic one-shot set + proposed verified VAE attribute augmented set with 1.2 *L*_2_ distance**	no overlap one-shot set	90.66	**90.99**	90.83	90.40	90.87
**basic one-shot set + image processing augmented set+ proposed verified VAE attribute augmented set with 1.2 *L*_2_ distance**	no overlap one-shot set	**95.83**	95.30	90.73	95.49	95.43

## Data Availability

Publicly available datasets were analyzed in this study. This data can be found here: http://vis-www.cs.umass.edu/lfw/ (accessed on 23 October 2021.)
